# Mining of simple sequence repeats (SSRs) loci and development of novel transferability-across EST-SSR markers from *de novo* transcriptome assembly of *Angelica dahurica*

**DOI:** 10.1371/journal.pone.0221040

**Published:** 2019-08-22

**Authors:** Chen Chen, Youjun Chen, Wenjuan Huang, Yijie Jiang, Huihui Zhang, Wei Wu

**Affiliations:** 1 Agronomy College, Sichuan Agricultural University, Chengdu, Sichuan Province, China; 2 Key Laborary of Superior Forage Germplasm in the Qinghai-Tibetan Plateau (2017-ZJ-Y12), Qinghai University, Xining, Qinghai Province, China; Chinese Academy of Medical Sciences and Peking Union Medical College, CHINA

## Abstract

*Angelica dahurica* is a widely grown plant species with multiple uses, especially in the medical field. However, the frequent introduction of *A*. *dahurica* to new areas has made it difficult to distinguish between varieties. Simple sequence repeats (SSRs) detected based on transcriptome analyses are very useful for constructing genetic maps and analyzing genetic diversity. They are also relevant for the molecular marker-assisted breeding of *A*. *dahurica*. We identified 33,724 genic SSR loci based on transcriptome sequencing data. A total of 114 primer pairs were designed for the SSR loci and were tested for their specificity and diversity. Ten SSR loci in untranslated regions were ultimately selected. Subsequently, 56 *A*. *dahurica* ecotypes collected from different regions were analyzed. The SSR loci comprised 2–8 alleles, with a mean of 5.2 alleles per locus. The polymorphic information content value and Shannon’s information index were 0.6274–0.2702 (average of 0.4091) and 1.3040–0.5618 (average of 0.8475), respectively. Thus, the 10 novel SSRs identified in this study were almost in accordance with Harvey-Weinberg equilibrium and will be useful for analyzing *A*. *dahurica* genetic relationships. The results of this study confirm the potential value of transcriptome databases for the development of new SSR markers.

## Introduction

*Angelica dahurica* [1, 2], which is known as ‘Baizhi’ in China, is a perennial dicotyledonous herb of the family *Apiaceae*. It originated in Taiwan and is widely grown in Korea, China, Japan, and Russia [3]. *A*. *dahurica* has been used in traditional Chinese medicine [4] because of its anti-proliferative [5], anti-inflammatory [6], anti-depressive [7], anti-oxidative [8], and anti-microbial [9] effects. It has also been applied to regulate hormones [10], decrease blood glucose levels [11], and treat headaches, ulcers, toothaches, abscesses [12], and other diseases. *Angelica dahurica* root extracts contain furanocoumarin compounds [13], phenolic compounds, and volatile oils, all of which account for the multiple pharmacological effects of this plant species. *A*. *dahurica* ecotypes may vary in their root surface texture and color, stem color, active ingredient compositions and abundances, and other characteristics. However, it remains unknown whether these differences are the result of phenotypic plasticity or ecotypic variation. Analyses of molecular data may clarify this issue.

*Angelica dahurica* has a functionally diploid genome (2*n* = 22) [14], and is cultivated in Sichuan, Zhejiang, Henan, Hebei, Zhejiang, Anhui, and other provinces in China. Additionally, there are many wild *A*. *dahurica* populations. The abundance of available *A*. *dahurica* germplasm resources is ideal for genetic studies as the core of germplasm resource diversity is genetic diversity. The development of molecular genetic markers may be useful for clarifying the genetic variation among varieties. Eukaryotic genes are broken up by introns [15]. In the process of eukaryotic gene expression, introns are spliced out to form different mRNAs that guide protein expression and ultimately produce phenotypic diversity. Therefore, SSR markers can be divided into two types: genic markers, such as those in mRNA coding regions and untranslated regions (UTRs), and genomic markers, including markers in non-functional genes and intron regions. SSR markers are usually developed through transcriptome sequencing. Compared with genomic markers, genic markers are more practical and may be closely associated with traits because variations in the nucleotide sequences of functional genes often result in phenotypic diversity.

Simple sequence repeats (SSRs), which are also known as microsatellites, are one of the most efficient types of genetic markers because of their reproducibility, multiallelic nature, codominant inheritance, relative abundance, and high genome coverage[16]. SSR loci are generally divided into genomic SSRs and genic SSRs based on their genomic locations. Genic SSRs occur in gene coding regions, making them suitable for explaining the phenotypic and functional diversity in various populations, gene mapping, and analyses of evolution [17].

The cross-species transferability of SSRs, which is dependent on phylogenetic closeness, is greater for genic SSRs than for genomic SSRs. Advances in next-generation (i.e., high-throughput) sequencing technology have improved the efficiency and reliability of transcriptome sequencing experiments[18]. Consequently, many expressed sequence tag (EST)-SSR markers exhibiting cross-species transferability have been developed via the transcriptome sequencing of various plant species, such as Euphorbiaceae family members [19], *Lolium multiflorum* [20], and *Saccharina japonica* [21]. The frequent introduction of *A*. *dahurica* to new regions in China has made it difficult to distinguish between different populations, which is problematic for the development of *A*. *dahurica*-specific EST-SSR markers. Thus, *A*. *dahurica*-specific EST-SSR markers have not been reported. We herein present the first report of the development of genic SSR markers in *A*. *dahurica*. These markers may provide researchers with new opportunities for assessing the molecular phylogeny and genetic diversity among *A*. *dahurica* varieties.

## Materials and methods

### 2.1 Plant materials

Our research object was *A*. *dahurica*, which is not an endangered or protected species. No specific permission was required for the materials we collected because they are ecotypes that have been planted for a long period, and the exchange of planting areas occurs frequently. We collected them with the owners’ permission. We collected 56 *A*. *dahurica* ecotypes from Sichuan, Zhejiang, Hebei, Jiangsu, Anhui, and Guizhou provinces as well as the Chongqing municipality (Table 1) for the subsequent development of genic SSR markers.

**Table 1:** 56 *A. dahurica* ecotypes collected from different regions.

No.	Material ID	Types	Origins	No.	Material ID	Types	Origins
1	1801	cultivar	Suining city, Sichuan province, China	29	BZC035	cultivar	Suining city, Sichuan province, China
2	1802	cultivar	Suining city, Sichuan province, China	30	BZC036	cultivar	Suining city, Sichuan province, China
3	1804	cultivar	Ziyang city, Sichuan province, China	31	BZC037	cultivar	Suining city, Sichuan province, China
4	1805	cultivar	Ziyang city, Sichuan province, China	32	BZC040	cultivar	Dazu district, Chongqing municipality, China
5	1806	cultivar	Nanchong city, Sichuan province, China	33	BZC048	cultivar	Suining city, Sichuan province, China
6	1807	cultivar	Luzhou city, Sichuan province, China	34	BZC052	cultivar	Suining city, Sichuan province, China
7	1808	cultivar	Suining city, Sichuan province, China	35	BZC056	cultivar	Suining city, Sichuan province, China
8	1809	cultivar	Suining city, Sichuan province, China	36	BZC057	cultivar	Suining city, Sichuan province, China
9	1810	cultivar	Suining city, Sichuan province, China	37	BZC059	cultivar	Suining city, Sichuan province, China
10	1811	cultivar	Suining city, Sichuan province, China	38	BZC060	cultivar	Suining city, Sichuan province, China
11	1812	cultivar	Neijiang city, Sichuan province, China	39	BZC061	cultivar	Suining city, Sichuan province, China
12	1814	cultivar	Dazhou city, Sichuan province, China	40	BZC062	cultivar	Suining city, Sichuan province, China
13	1815	cultivar	Guang’an city, Sichuan province, China	41	BZC063	cultivar	Suining city, Sichuan province, China
14	1816	cultivar	Guang’an city, Sichuan province, China	42	BZC064	cultivar	Suining city, Sichuan province, China
15	1817	cultivar	Zunyi city, Guizhou province, China	43	BZC067	cultivar	Suining city, Sichuan province, China
16	BZA001	cultivar	Bred by Sichuan Agricultural University	44	BZC069	cultivar	Dazu district, Chongqing municipality, China
17	BZA002	cultivar	Bred by Sichuan Agricultural University	45	BZC070	cultivar	The specimen garden of Sichuan Agricultural University
18	BZA003	cultivar	Bred by Sichuan Agricultural University	46	BZC071	cultivar	Suining city, Sichuan province, China
19	BZA004	cultivar	Bred by Sichuan Agricultural University	47	BZC072	cultivar	Dazu district, Chongqing municipality, China
20	BZB002	cultivar	Bred by Sichuan Agricultural University	48	BZC073	cultivar	Suining city, Sichuan province, China
21	BZB003	cultivar	Bred by Sichuan Agricultural University	49	BZC074	cultivar	Suining city, Sichuan province, China
22	BZC014	cultivar	Suining city, Sichuan province, China	50	BZC079	cultivar	Suining city, Sichuan province, China
23	BZC015	cultivar	Suining city, Sichuan province, China	51	BZC080	cultivar	Suining city, Sichuan province, China
24	BZC017	cultivar	Nanchong city, Sichuan province, China	52	BZC081	cultivar	Anhui province, China
25	BZC018	cultivar	Anguo city, Hebei province, China	53	BZC082	cultivar	Sichuan province, China
26	BZC019	cultivar	Suining city, Sichuan province, China	54	BZC084	cultivar	Jiangsu province, China
27	BZC024	cultivar	Suining city, Sichuan province, China	55	BZC086	cultivar	Hangzhou city, Zhejiang province, China
28	BZC033	cultivar	Suining city, Sichuan province, China	56	BZC089	cultivar	Anguo city, Hebei province, China

Leaves, stems, and phloem and xylem tissues of *A*. *dahurica* cultivar ‘Chuan zhi No. 2’ were collected at the seedling and bolting stages for transcriptome sequencing. The samples were immediately frozen in liquid nitrogen and stored at −80°C for subsequent RNA extraction. Three biological replicates were prepared for each sample.

### 2.2 RNA extraction, sequencing, and analysis of the *A*. *dahurica* transcriptome and SSR locus identification

Total RNA was extracted with the mirVana miRNA ISOlation Kit (Thermo Fisher, Carlsbad, CA, USA). The 2100 Bioanalyzer RNA Nanochip (Agilent, Santa Clara, CA, USA) was used to verify RNA quality. All samples had an RNA Integrity Number > 7 and a 28S/18S ratio > 2. The TruSeq Stranded mRNA LT Sample Prep Kit (Illumina, San Diego, CA, USA) was used to purify and fragment the mRNA, after which double-stranded cDNA was synthesized with SuperScript II Reverse Transcriptase (Invitrogen, Carlsbad, CA, USA). Short fragments were modified with an A-tail on the 3′ end and adapters were ligated via PCR amplification. The resulting modified fragments were sequenced with the Illumina Genome Analyzer HiSeq X Ten sequencing platform. Data analyses and base calling were performed with the Illumina instrument software. The transcriptome datasets are available in the NCBI Sequence Read Archive (accession number SRX247927).

The Trinity software (version: trinityrnaseq_r20131110) [1] was used to splice and obtain transcript sequences. Additionally, the MISA software (http://pgrc.ipk-gatersleben.de/misa/) was used to detect genic SSR markers based on the *de novo* transcriptome sequencing data [22]. The SSR loci containing repeat units of 1–6 nucleotides were identified, and the minimum SSR length was set at six iterations. SSR primers were designed using the Primer3 software (http://pgrc.ipk-gatersleben.de/misa/) with the following parameters: primer length of 18–22 bp, with an optimum length of 20 bp; an annealing temperature of 60°C; a PCR product size of 100–280 bp; and a GC content of 40–60%, with an optimum of 50% [23].

### 2.3 Germplasm resource DNA extraction

The seeds of the various ecotypes were sown in pots and germinated in a growth chamber under the following conditions: 22°C, 75% relative humidity, and a 16-h light/8-h dark cycle. Leaves were collected at the seedling stage, and genomic DNA was extracted according to a modified cetyltrimethylammonium bromide protocol, with polysaccharides removed using high salt concentrations and polyphenols removed using polyvinyl pyrrolidone, an extended RNase treatment and phenol-chloroform extraction [24]. The DNA quality was assessed by 1% agarose gel electrophoresis (m/v) and OD260/OD280 with a NanoDrop 2000 spectrophotometer (Wilmington, DE, USA). The DNA quantity was assessed with a NanoDrop 2000 spectrophotometer.

### 2.4 Development and validation of SSR markers

A total of 114 SSR primer pairs were synthesized by Chengdu TsingKe Biotechnology Co., Ltd. (Chengdu, China). PCR amplifications were completed with the T100 Thermal Cycler (Foster City, CA, USA). The PCR program was as follows: 94°C for 5 min; 35 cycles of 94°C for 30 s, 55–65°C for 30 s, and 72°C for 2 min; 72°C for 10 min. The specificity and ideal annealing temperature of the PCR products were determined by 1% agarose gel electrophoresis. Primers that amplified a single band of the expected size were selected.

The forward primers were labeled with HEX fluorescent probe by Chengdu TsingKe Biotechnology Co., Ltd. (Chengdu, China) to analyze fragments on the Applied Biosystems 3730xl DNA Analyzer (Carlsbad, CA, USA). For genotyping, 0.5 μL PCR amplification products in 10 µL ABI highly deionized (Hi-Di) formamide and ABI GeneScan 500 LIZ Size standard mixture (130:1) were first pre-degenerated at 95℃ for 5 min and then separated by capillary electrophoresis. Alleles were detected with the GeneMapper software (version 4.1) [25]. Markers with a strong tendency to form stutter peaks were excluded in this step. The sequences of SSR products were determined by Chengdu TsingKe Biotechnology Co., Ltd. (Chengdu, China).

### 2.5 Data analysis

We used the GenAlEx software (version 6.501) [26] to edit the data and transform it the codominant format automatically. The number of alleles (Na), number of effective alleles (Ne), observed heterozygosity (Ho), expected heterozygosity (He), Shannon’s information index (I), and Hardy-Weinberg equilibrium (HWE) were calculated with the Popgene software (version 1.32) [27]. Additionally, the polymorphic information content (PIC) was calculated with the PIC_Calc program.

## Results

### 3.1 Transcriptome sequencing and *de novo* assembly

**Fig 1:**
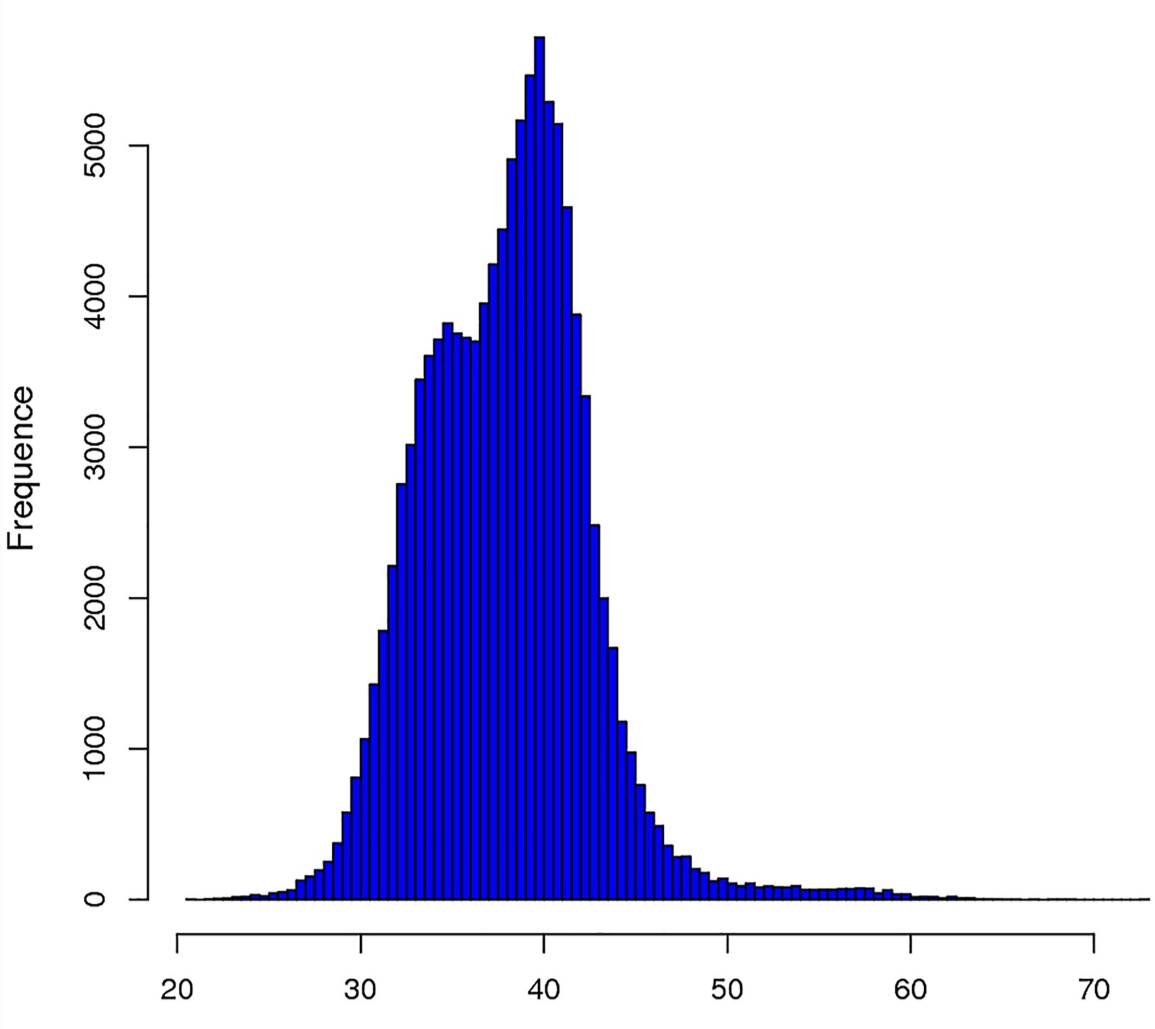
GC Content frequence distribution of unigene.

The quality of the extracted total RNA was appropriate for transcriptome sequencing (see S1 Table, S1 Fig). After a stringent quality assessment and data filtering step, 49,580,458.67 raw reads were selected for further analyses and deposited in the NCBI SRA database (PRJNA523076). An overview of the sequencing results is presented in Table 2. All high-quality reads were assembled with the Trinity software, which produced a contig N50 length of 1,858 bp and a mean length of 1,299.83 bp. After sequencing, 110,251 unigenes were identified in the *A*. *dahurica* transcriptome based on the *de novo* assembly of clean raw reads comprising a total length of 143,307,954 bp. The GC content of the unigenes was mostly 30–50% (Fig 1). The mean *A*. *dahurica* transcriptome GC content was 43.33%.

**Table 2:** Transcriptome sequencing data quality and splicing results statistics.

Projects	Numbers	Projects	Numbers
Raw reads	49580458.67	Total number of Unigene >300 (bp)	110251
Raw bases	7437068800.00	Total number of Unigene≥500 (bp)	86958
Clean reads	47550772.74	Total number of Unigene≥1000 (bp)	50559
Clean bases	7128569462.00	N50	1858
Valid bases	95.86%	The total length of unigene (bp)	143307954
Q30	91.97%	The length of the longest unigene (bp)	20496
GC	43.33%	The length of the shortest unigene (bp)	290
Raw Bases(G)	6.93	The average length of unigene (bp)	1299.83
Clean bases(G)	6.64		

### 3.2 Identification of simple sequence repeats from the *A*. *dahurica* transcriptome

A total of 33,724 potential SSR loci were identified across 26,455 unigenes from the transcriptome of *A*. *dahurica*. The total size of the examined unigene sequences was 143,307,954 bp, meaning one SSR site was detected per 4,249 bp. Moreover, 20,734 unigenes (78.37%) contained a single SSR, 5,723 unigenes (21.63%) had more than one SSR, and 1,993 unigenes (7.53%) contained compound SSRs (Table 3). Thus, *A*. *dahurica* contains many types of SSRs, and all of the repeats comprising 1–6 nucleotides were detected.

**Table 3:** *A*. *dahurica* transcriptome SSR general statistics.

Items	Number
Total number of unigene sequences examined	110251
Total size of examined unigene sequences (bp)	143307954
Total number of identified SSRs	33724
The number of sequences containing SSR loci	26455
The number of sequences with 1 SSR loci	20734
The number of unigene sequences with more than 1 SSR locus	5723
The number of SSRs present in compound formation	1993

**Fig 2:**
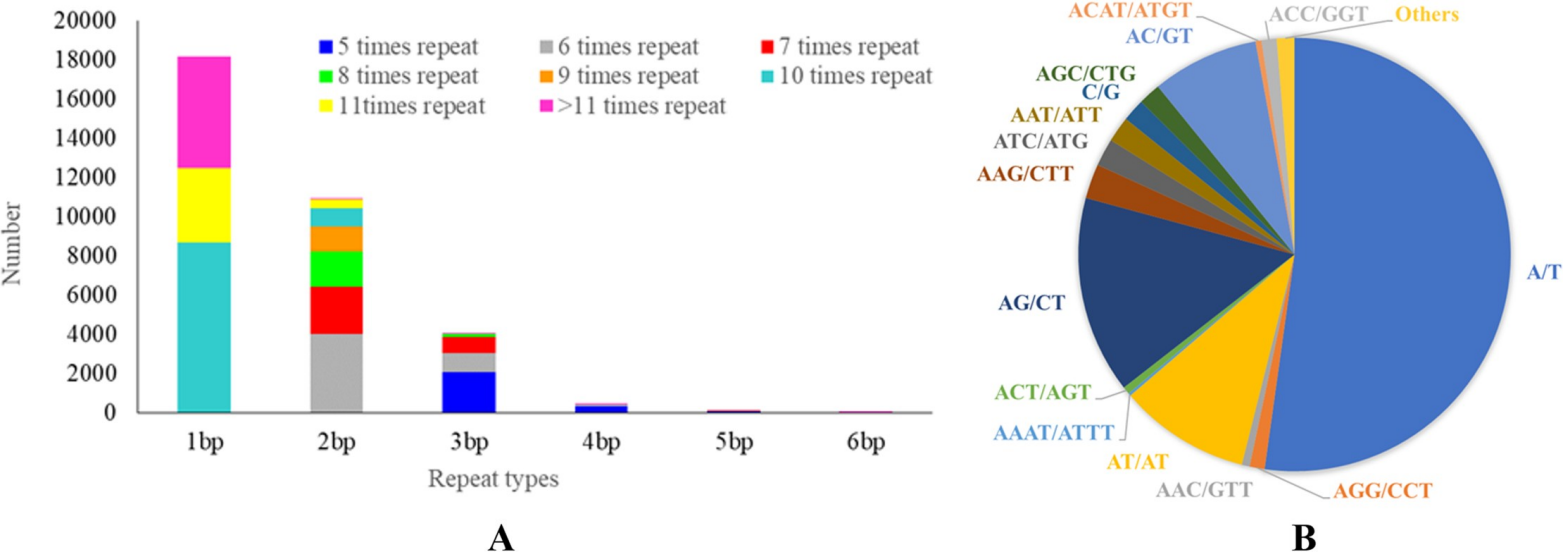
Repeat motif type distribution in *A*. *dahurica* SSR. A:1–6 nucleotides repeats statistical results, B: types and quantities of dinucleotides and trinucleotide repeats.

In total, 18,179 mononucleotide repeat motifs with more than 10 repeats (53.91%) were identified, which was the most frequent type, followed by dinucleotide repeat motifs with more than six repeats (10,975; 32.54%), trinucleotide repeat motifs with more than five repeats (4,022; 11.93%), tetranucleotide repeat motifs with more than five repeats (436; 1.29%), pentanucleotide repeat motifs with more than five repeats (77; 0.23%), and hexanucleotide repeat motifs with more than five repeats (35; 0.1%). Additionally, A/T was the most abundant motif among the two possible types of mononucleotide repeats, representing 52.51% of the total SSRs, followed by the AG/CT (14.73%), AT/AT (9.80%), and AC/GT (7.99%) motifs. Among the trinucleotide repeat motifs, the two most frequent repeats were AAG/TTC (2.58% of the total SSRs) and ATC/ATG (2.06%), followed by AAT/ATT (1.92%) and AGC/CTG (1.66%) (Table 4). Quad-, penta-, and hexanucleotide repeats represented 1.62% of the total SSRs (Fig 2A).

**Table 4:** The most abundant SSR repeat types in the transcriptome.

Mononucleotide	Dinucleotide	Trinucleotide	Tetranucleotide
A/T (52.21%)	AG/CT (14.73%)	AAG/CTT (2.58%)	ACAT/ATGT (0.45%)
C/G (1.69%)	AT/AT (9.80%)	ATC/ATG (2.06%)	AAAT/ATTT (0.20%)
	AC/GT (7.99%)	AAT/ATT (1.92%)	AGAT/ATCT (0.10%)
	CG/CG (0.03%)	AGC/CTG (1.66%)	AAAG/CTTT (0.07%)
		ACC/GGT (1.13%)	AGGG/CCCT (0.07%)
		AGG/CCT (1.13%)	AAAC/GTTT (0.06%)
		AAC/GTT (0.59%)	AGCT/AGCT (0.05%)
		ACT/AGT (0.56%)	AACC/GGTT (0.04%)
		ACG/CGT (0.18%)	ACAG/CTGT (0.03%)
		CCG/CGG (0.13%)	ACTC/AGTG (0.03%)
			AGGC/CCTG (0.03%)

### 3.3 Development and validation of identified SSR markers

A total of 114 primer pairs were designed and synthesized (S1 Table). After PCR amplification, 14 primer pairs that produced a single band of the expected size and many polymorphisms were selected for subsequent analysis. All of the forward primers were labeled with different HEX fluorescent dyes and then used to analyze the fragments from the 56 *A*. *dahurica* ecotypes collected from different locations in China (Table 1). Loci that failed to provide clear signals in the expected size range or that lacked polymorphisms were eliminated. Finally, 10 novel SSR primer pairs were developed for *A*. *dahurica* (Table 5). A total of 45 alleles were identified, with 2–8 alleles per locus. The observed and expected heterozygosities at each locus were 0.6955–0.3137 (average of 0.5332) and 0.6802–0.3018 (average of 0.4626), respectively. Shannon’s information index was 1.3040–0.5618 (average of 0.8475) and the PIC was 0.6274–0.2702 (average of 0.4091). The loci were moderately polymorphic. HWE tests revealed that only Locus 8 significantly deviated from HWE in this captive population (0.05 ≥ *P* ≥ 0.01). The results showed Loci 1, 2, 3, 4, 7, 9, and 10 were moderately polymorphic and in accordance with HWE.

**Table 5:** Characteristics of the 10 polymorphic SSR markers and the novel microsatellite marker system validated in 56 *A*. *dahuricaes*.

loci No.	Motif	Primer Sequence(5'→3')		Tm (℃)	Allele Size (bp)	Na	Ne	Ho	He	I	PIC	HWE	Regions
1	(TA)8	F:	GCCTCAAGAACTTGCCTCAC	61	229–241	4	1.4323	0.6955	0.3018	0.5618	0.2702	0.1328	5’UTR
		R:	GTTTCACCACTTCTGCCACA										
2	(AG)9	F:	CAGACCTGAATGGCACTGAA	60	173–181	6	1.8518	0.5359	0.46	0.9361	0.4292	0.3709	5’UTR
		R:	TGAAGGAGTCTGCACACCAA										
3	(TG)7	F:	GATTGAGCAAGGAGAGGTGC	60	241–250	4	1.7398	0.5709	0.4252	0.7959	0.3835	0.3024	5’UTR
		R:	CCATCAAATAAGAGGCACAAAA										
4	(CA)6	F:	CAGCCGGCTAAGACAGAATC	60	254–258	2	1.6228	0.6128	0.3838	0.5719	0.3101	0.2457	5’UTR
		R:	TGGGATCACTTTCTTGGCATA										
5	(TA)7	F:	TTTTCATGGATGCACAATAAGC	57	200–210	6	3.1266	0.3137	0.6802	1.304	0.6272	0.1465	5’UTR
		R:	GAAATTGATGGCATTCTCCC										
6	(TC)9	F:	TTGCAGCGTCAATTTGCTAC	59	237–243	5	2.48	0.3978	0.5968	1.1452	0.5498	0.2736	5’UTR
		R:	AGAAAAAGTCCGGGCAAAGT										
7	(AT)8	F:	TTACGCAATTGCTGATTCCA	58	212–220	3	1.9222	0.5159	0.4798	0.7084	0.3729	0.3362	5’UTR
		R:	CCCCGCTGGCTTATCTTTAT										
8	(AT)9	F:	CCATGGAGAAATTGGCTGTT	58	149–158	8	1.8909	0.5246	0.4711	1.0793	0.4521	0.0102	5’UTR
		R:	TTCTTGATCCAGTGATGCCA										
9	(GT)9	F:	TTTGGGGCTGCTTTGTATTC	59	269–285	3	1.7302	0.5742	0.422	0.6872	0.3528	0.6730	5’UTR
		R:	TCCAGCATCCAGTATCACCA										
10	(TG)9	F:	GGTTTGTGAGATCCTTTGGG	60	237–243	4	1.682	0.5909	0.4055	0.6852	0.3427	0.6662	5’UTR
		R:	GAACTGACACTTGGGAATCCA										

Na: Number of Different Alleles, Ne: Number of Effective Alleles, I: Shannon‘s Information Index, Ho: Observed Heterozygosity, He: Expected Heterozygosity, PIC: Polymorphism Information Content. HWE: Hardy-Weinberg equilibrium P-value (≥0.05).

## Discussion

### 4.1 Developing SSRs based on the transcriptome is reliable and efficient

With the advent and rapid development of high-throughput sequencing technology, RNA sequencing and the development of molecular markers has become easy and reliable in many species [28–30]. Multiple SSR loci can be identified by analyzing the transcriptome, which is significant for the localization of genes responsible for specific traits and functions. This is the first report describing the application of high-throughput sequencing to the development of *A*. *dahurica* genic SSR markers based on unigenes. A total of 33,724 genic SSR loci were detected, and a set of 10 novel genic SSR markers was developed to provide additional tools for analyzing the genetic diversity of *A*. *dahurica*. The 10 polymorphic loci were then validated in 56 individuals. These loci showed abundant polymorphism, implying they are useful for analyzing *A*. *dahurica* relationships and confirming the potential value of an *A*. *dahurica* transcriptome database for the development of new SSR markers.

### 4.2 The infrequent observation of GC repeats may be related to CpG clusters and GC contents

GC repeat motifs were the least common among the mononucleotide and dinucleotide repeats, possibly because of the presence of CpG clusters. Previous studies have indicated that CpG dinucleotides often occur in discrete regions, and almost 60% of these CpG-rich clusters are located in or close to genes, mostly at the 5′ end [31, 32]. The GC content and CpG pattern, along with the chromatin condensation and B–Z transition, in a gene and around the promoter can determine gene expression characteristics through transcription factor binding sites [33]. Cell differentiation involving the deamination of mC to T has been proposed to result in CpG deficiency [34], and CpG deficiency is associated with a corresponding TpG (CpA) excess [35]. The mean GC content of the *A*. *dahurica* transcriptome unigenes was 43.33% (i.e., less than the AT content), which may be related to the limited number of GC dinucleotide repeats or G/C mononucleotides. Moreover, CpG clusters may be essential for regulating gene expression, and are therefore infrequently observed.

### 4.3 The presence of 10 novel SSRs in untranslated regions implies SSR diversity is related to different phenotypes

*Cis*-acting elements involved in post-transcriptional control are generally located in the UTRs of mRNAs [36]. In the current study, 10 novel *A*. *dahurica* SSRs were localized to UTRs. UTRs are vital at the interface between mRNAs and proteins. They regulate stability, transport, and translation efficiency, as well as the function and subcellular localization of translated proteins, and they increase the coding capacity of the genome [36–39]. In future studies, the relationship between SSRs and regulatory elements in the UTR should be characterized to clarify phenotypic diversity.

## Conclusion

The *A*. *dahurica* transcriptome characterization and the substantial body of transcripts reported here will facilitate research to develop the medicinal and nutritional properties of this species. Ten novel *A*. *dahurica* SSR markers with polymorphism were developed, which provides a foundation for genetic diversity analysis, genetic mapping and marker breeding in *A*. *dahurica*.

## Supporting information

S1 Fig RNA quality check of *A. dahurica* samples. (DOCX)

S1 Table RNA detection of *A. dahurica* samples. (DOCX)

S2 Table 114 pairs primers sequence and SSR loci analysis. (XLSX)
