# Supplementary material for: Mining of simple sequence repeats (SSRs) loci and development of novel transferability-across EST-SSR markers from de novo transcriptome assembly of Angelica dahurica
Source: PLoS One. 2019 Aug 22;14(8):e0221040. doi: 10.1371/journal.pone.0221040 (PMC6706007; doi:10.1371/journal.pone.0221040)

**Supplementary Figure 1 1% agarose gel detection samples RNA quality**


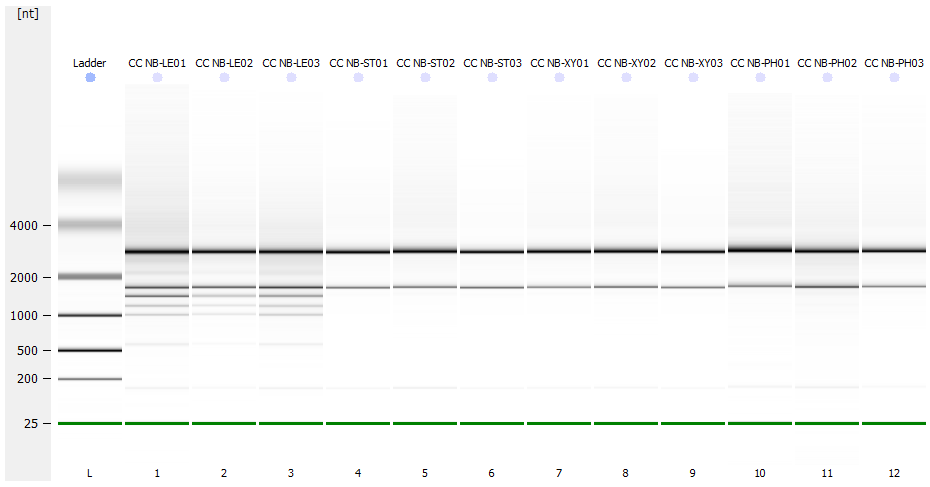

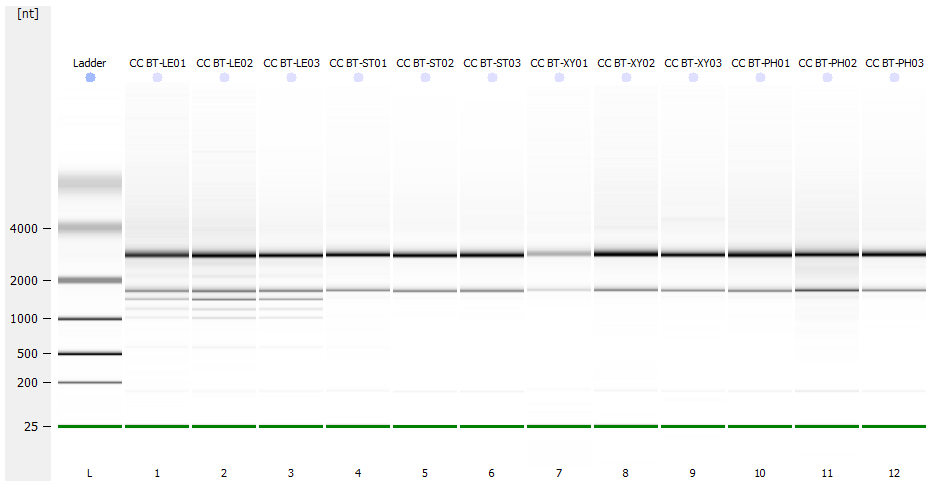


**Supplementary Figure 2 Capillary electrophoresis detection samples RNA quality**


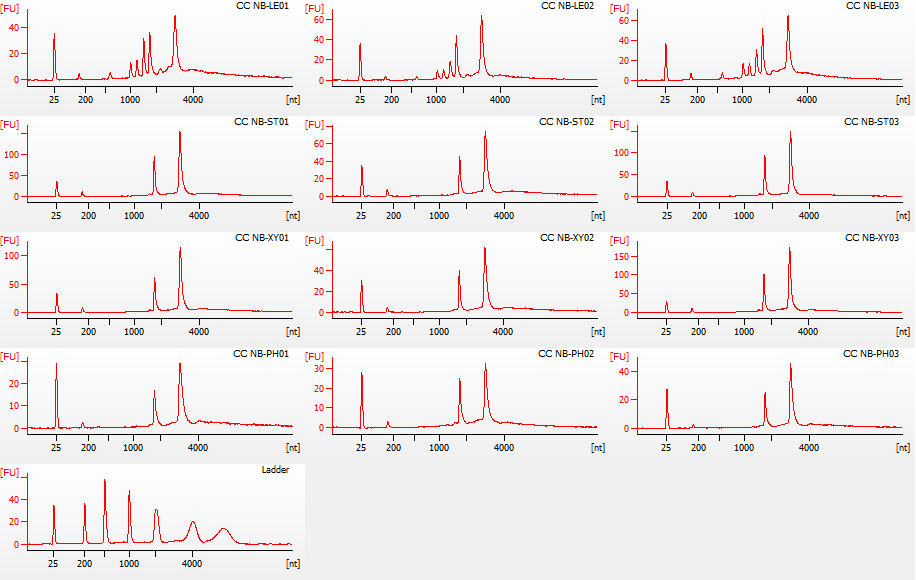

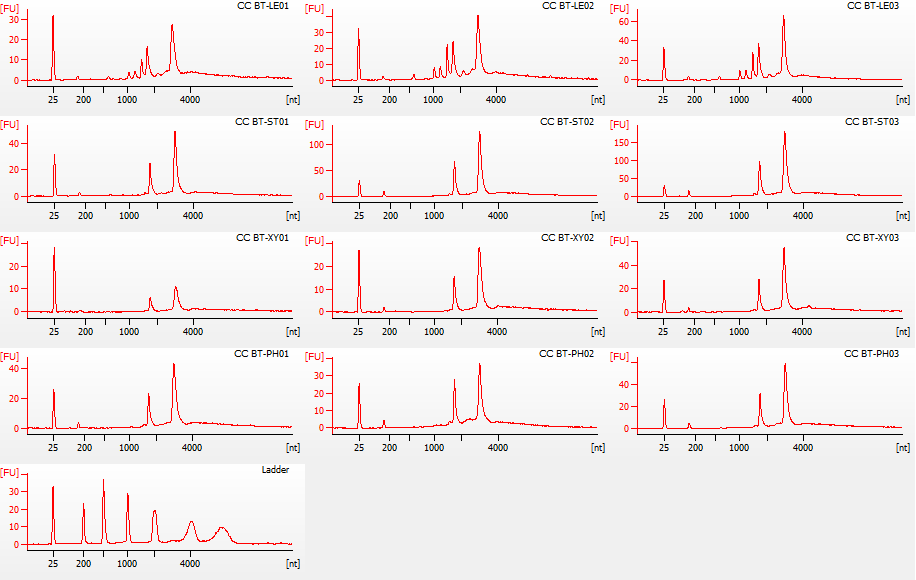

Supplement: S1 Fig — (DOCX) [file pone.0221040.s001.docx]
