# Supplementary material for: Mining of simple sequence repeats (SSRs) loci and development of novel transferability-across EST-SSR markers from de novo transcriptome assembly of Angelica dahurica
Source: PLoS One. 2019 Aug 22;14(8):e0221040. doi: 10.1371/journal.pone.0221040 (PMC6706007; doi:10.1371/journal.pone.0221040)
Supplement: S1 Table — (DOCX) [file pone.0221040.s002.docx]

Supplementary table 1 RNA detection of Angelica dahurica

| Sample No. | RNA Concentration（μg/μl） | A260/280 | A260/230 | 28S/18S | RIN | Result |
| --- | --- | --- | --- | --- | --- | --- |
| se-full-1 | 0.5110 | 2.22 | 2.23 | 2.3 | 7.2 | Meet sequencing requirements |
| se-full-2 | 0.5618 | 2.25 | 2.21 | 2.4 | 7.6 |  |
| se-full-3 | 0.7009 | 2.23 | 2.05 | 2.4 | 7.8 |  |
| NB-LE01 | 0.7170 | 2.03 | 1.25 | 2.4 | 7.9 |  |
| NB-LE02 | 0.6425 | 2.03 | 1.81 | 2.4 | 8.5 |  |
| NB-LE03 | 0.9330 | 2.17 | 2.23 | 2.1 | 7.6 |  |
| NB-ST01 | 0.8846 | 2.17 | 2.37 | 2.3 | 10.0 |  |
| NB-ST02 | 0.7579 | 2.01 | 1.98 | 2.5 | 10.0 |  |
| NB-ST03 | 1.0351 | 2.16 | 2.38 | 2.1 | 9.9 |  |
| NB-XY01 | 0.7575 | 2.00 | 2.28 | 2.5 | 10.0 |  |
| NB-XY02 | 1.0655 | 2.17 | 2.10 | 2.6 | 10.0 |  |
| NB-XY03 | 0.8463 | 2.17 | 2.24 | 2.3 | 9.8 |  |
| NB-PH01 | 0.1374 | 2.11 | 1.95 | 2.8 | 10.0 |  |
| NB-PH02 | 0.3612 | 2.16 | 2.16 | 2.2 | 9.3 |  |
| NB-PH03 | 0.1803 | 2.13 | 1.60 | 2.9 | 9.9 |  |
| BT-LE01 | 0.3932 | 2.16 | 2.08 | 2.9 | 8.8 |  |
| BT-LE02 | 0.5401 | 2.18 | 2.06 | 2.3 | 8.2 |  |
| BT-LE03 | 0.5764 | 2.18 | 2.12 | 2.6 | 8.6 |  |
| BT-ST01 | 0.2738 | 2.18 | 1.69 | 2.9 | 9.7 |  |
| BT-ST02 | 0.7237 | 2.06 | 2.20 | 2.5 | 10.0 |  |
| BT-ST03 | 0.8448 | 2.07 | 2.27 | 2.5 | 10.0 |  |
| BT-XY01 | 0.1588 | 2.11 | 1.78 | 2.5 | 10.0 |  |
| BT-XY02 | 0.2672 | 2.16 | 1.47 | 2.5 | 10.0 |  |
| BT-XY03 | 0.3785 | 2.16 | 1.09 | 2.9 | 10.0 |  |
| BT-PH01 | 0.3984 | 2.15 | 1.71 | 3.3 | 10.0 |  |
| BT-PH02 | 0.4444 | 2.03 | 2.10 | 2.0 | 8.8 |  |
| BT-PH03 | 0.4580 | 2.01 | 1.39 | 2.7 | 10.0 |  |

RNA quality criteria: RIN>=7且28S/18S>=0.7
